# *Neisseria meningitidis* Uses Sibling Small Regulatory RNAs To Switch from Cataplerotic to Anaplerotic Metabolism

**DOI:** 10.1128/mBio.02293-16

**Published:** 2017-03-21

**Authors:** Yvonne Pannekoek, Robert A. G. Huis in ‘t Veld, Kim Schipper, Sandra Bovenkerk, Gertjan Kramer, Matthijs C. Brouwer, Diederik van de Beek, Dave Speijer, Arie van der Ende

**Affiliations:** a Department of Medical Microbiology, Center for Infection and Immunity (CINIMA), Academic Medical Center, Amsterdam, The Netherlands; b Department of Medical Biochemistry, Academic Medical Center, Amsterdam, The Netherlands; c Department of Neurology, Amsterdam Neuroscience, Academic Medical Center, Amsterdam, The Netherlands; The Sanger Institute

**Keywords:** *Neisseria meningitidis*, metabolic regulation, pathogenesis, sibling sRNAs, small regulatory RNAs, stringent response

## Abstract

*Neisseria meningitidis* (the meningococcus) is primarily a commensal of the human oropharynx that sporadically causes septicemia and meningitis. Meningococci adapt to diverse local host conditions differing in nutrient supply, like the nasopharynx, blood, and cerebrospinal fluid, by changing metabolism and protein repertoire. However, regulatory transcription factors and two-component systems in meningococci involved in adaptation to local nutrient variations are limited. We identified novel sibling small regulatory RNAs (*Neisseria* metabolic switch regulators [NmsRs]) regulating switches between cataplerotic and anaplerotic metabolism in this pathogen. Overexpression of NmsRs was tolerated in blood but not in cerebrospinal fluid. Expression of six tricarboxylic acid cycle enzymes was downregulated by direct action of NmsRs. Expression of the NmsRs themselves was under the control of the stringent response through the action of RelA. Small sibling regulatory RNAs of meningococci, controlling general metabolic switches, add an exciting twist to their versatile repertoire in bacterial pathogens.

## IMPORTANCE

Regulatory small RNAs (sRNAs) of pathogens are coming to be recognized as highly important components of riboregulatory networks, involved in the control of essential cellular processes. They play a prominent role in adaptation to physiological changes as represented by different host environments. They can function as posttranscriptional regulators of gene expression to orchestrate metabolic adaptation to nutrient stresses. Here, we identified highly conserved sibling sRNAs in *Neisseria meningitidis* which are functionally involved in the regulation of gene expression of components of the tricarboxylic acid cycle. These novel sibling sRNAs that function by antisense mechanisms extend the so-called stringent response which connects metabolic status to colonization and possibly virulence as well as pathogenesis in meningococci.

## INTRODUCTION

*Neisseria meningitidis* causes meningitis and septicemia with a high case fatality ratio (1) but normally resides innocuously in the nasopharynx of humans. To cause disease, the meningococcus has to pass the nasopharyngeal epithelium, enter the bloodstream to cause sepsis, and subsequently cross the blood-brain barrier to cause meningitis. The different compartments encountered can be regarded as separate environments with different nutrient supplies requiring adaptation of the meningococcal metabolism (2). The bacterial reorganization of cellular transcription (and thus gene expression) upon environmental changes, such as starvation and hypoxia, is referred to as the stringent response (3, 4). This response is mediated by the alarmones guanosine 5′,3′-bispyrophosphate and guanosine pentaphosphate, ppGpp and pppGpp, collectively referred to here as (p)ppGpp (5). In *Escherichia coli*, (p)ppGpp is synthesized from GTP and ATP via the action of two paralogous enzymes, RelA and SpoT (4, 6). The transcriptional changes occur mainly as a result of the direct effects of (p)ppGpp and its cofactor, the (protein) transcription factor DksA, on RNA polymerase (4). In addition to (p)ppGpp and regulatory proteins, among which are the transcription factors (TFs) small regulatory RNAs (sRNAs) are also involved in the switch from nutrient-rich (feast) to nutrient-limiting (famine) growth conditions of bacteria (7, 8).

sRNAs are important players in many cellular processes and prominent in those involving adaptive physiological changes. They can function as posttranscriptional regulators of gene expression to orchestrate stress responses and metabolism. Many sRNAs are synthesized upon nutritional stresses encountered by pathogens. They often regulate expression of target mRNAs that form part of a single nutritional regulatory circuit or network. sRNAs usually act by occupying or freeing up ribosomal entry sites of target transcripts as well as by regulating the accessibility of transcripts for RNases in an antisense fashion (9–12). The RNA chaperonin protein Hfq is frequently involved, enhancing these processes (13, 14).

We identified two highly conserved sRNAs, designated sibling *Neisseria* metabolic switch regulators (NmsRs), in *N. meningitidis* which are functionally involved in the regulation of tricarboxylic acid (TCA) cycle activity by antisense mechanisms. These novel sibling sRNAs extend the stringent response in meningococci, thereby connecting metabolic status to colonization and, possibly, virulence.

## RESULTS

**FIG 1:**
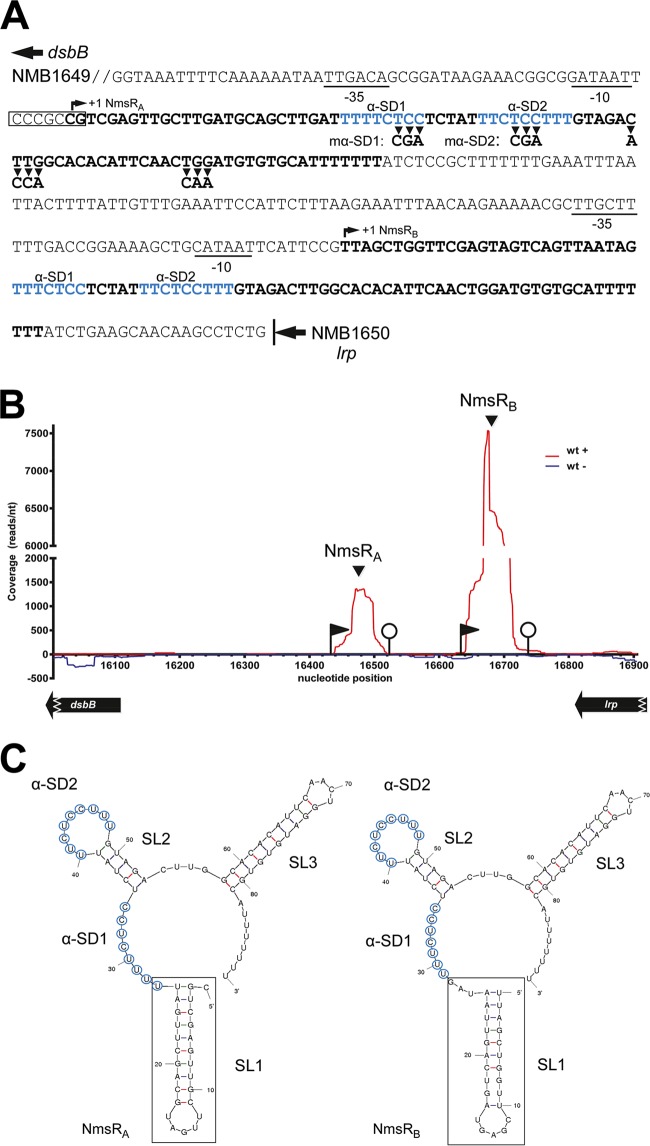
Primary sequence, expression analysis, and predicted secondary structures of NmsRs. (A) Sequence and genomic localization of NmsR_A_ and NmsR_B_ genes in *Neisseria meningitidis*. Transcriptional starts (TSSs) of NmsR_A_ and NmsR_B_, as determined by WTA, are indicated by +1. Putative promoter sequences (−35 and −10 boxes) are underlined. The GC-rich region between the −10 box and the TSS of NmsR_A_ is boxed. Transcribed regions are indicated in bold. Predicted anti-Shine-Dalgarno (SD) sequence motifs (α-SD1 and α-SD2) are shown in blue; nucleotide substitutions in α-SD1 (mα-SD1) (TCC^32−34^→CGA^32−34^) and α-SD2 (mα-SD2) (TCC^43−45^→CGA^43−45^) of NmsR_A_ and outside α-SD regions (CTTG^54−57^→ACCA^54−57^ and TGG^71−73^→CAA) are indicated by arrowheads. (B) Read coverage visualization of the expression of NmsR_A_ and NmsR_B_ in H44/76. Transcription on the plus strand is visualized on the positive *x* axis in red; transcription on the minus strand is visualized on the negative *x* axis in blue. Nucleotide position refers to contig 28 of H44/76 WGS (AEQZ01000018.1). The black flags indicates TSSs; the white circles indicate predicted Rho-independent terminators. Note that the region visualized encodes two distinct transcripts, indicated as NmsR_A_ and NmsR_B_. Coverage of the NmsR_B_ transcript is 5-fold (~7,500 reads/nt); that of the transcripts of NmsR_A_ is 1,500 reads/nt. (C) Predicted secondary structures of siblings NmsR_A_ and NmsR_B_. Secondary structures were predicted using Mfold. Unique sequences of the NmsRs are indicated by boxes. Putative α-SD sequences are circled; stem-loops are indicated as SL.

In whole-transcriptome analysis (WTA) of meningococci grown in nutrient-rich culture medium, we identified two structurally nearly identical sRNAs with 70% sequence identity (sibling sRNAs; NmsR_A_ and NmsR_B_), tandemly arranged in *N. meningitidis* strain H44/76 (Fig. 1) (15). Sequence read coverage of the NmsR_B_ transcript is 5-fold (~7,500 reads/nucleotide [nt]) that of NmsR_A_ (~1,500 reads/nt) (Fig. 1B). Among 7,335 isolates, 16 NmsR_A_ alleles with 14 single nucleotide polymorphisms (SNPs) were observed, with 97% of the isolates sharing two alleles with only one SNP. In addition, 19 NmsR_B_ alleles with 17 SNPs were observed, with 94% of the isolates sharing two alleles with only 4 SNPs (assessed at http://pubmlst.org/neisseria/) (16), and were located in the intergenic region in the reference *N. meningitidis* MC58 genome between locus NMB1649 (*dsbB*), encoding disulfide bond formation protein B, and NMB1650 (*lrp*), encoding leucine-responsive regulatory protein (17) (Fig. 1).

### NmsR_A_ and NmsR_B_ overexpression impairs growth in CSF but not in blood.

**FIG 2:**
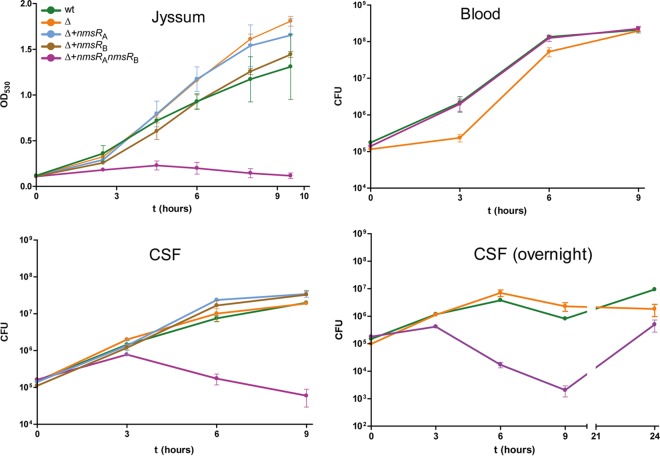
Overexpression of NmsRs leads to growth defects in minimal medium and CSF. Growth characteristics of different variant meningococci in Jyssum medium, blood, and CSF are shown. For growth in Jyssum medium, points represent means from two biological replicates (error bars show standard deviations); for growth curves in blood and CSF, points represent means from 5 technical replicates of one biological experiment. The different variant strains used are indicated in the figure (strain designations are explained in the legend to Fig. 4). Note that prolonged incubation (overnight) in CSF of meningococci overexpressing NmsRs results in escape variants (error bars show standard errors of the means).

To investigate the functionality of NmsRs in meningococci, we created an *nmsR*_A_- and *nmsR*_B_-knockout strain of H44/76 (the Δ*nmsR*_A_ Δ*nmsR*_B_ strain) by replacing *nmsR*_A_ and *nmsR*_B_ with an erythromycin (Erm) resistance cassette, and we introduced a plasmid harboring the genes encoding both NmsRs, NmsR_A_, or NmsR_B_ into the Δ*nmsR*_A_ Δ*nmsR*_B_ strain, thereby generating four variant meningococcal strains, one without the NmsRs and three variants overexpressing either NmsR_A_, NmsR_B_, or both in isogenic backgrounds. The effect of NmsR_A_ and NmsR_B_ expression was first assessed in meningococci grown under two culture conditions, tryptic soy broth (TSB) (nutrient rich) and Jyssum medium (glucose as the sole carbon source [18]). All four meningococcal variants and the wild-type (wt) strain replicated equally well in rich medium (GC or TSB) (not shown). The meningococcal strain overexpressing both NmsRs did not replicate in nutrient-poor Jyssum medium, while the wt strain, Δ*nmsR*_A_ Δ*nmsR*_B_, and the strains harboring only single *nmsR* plasmids grew normally (Fig. 2). *In vivo* relevance of NmsRs was shown by meningococcal culture in human blood or cerebrospinal fluid (CSF). Wild-type meningococci and meningococci overexpressing NmsRs showed similar growth in blood (Fig. 2). Meningococci overexpressing NmsRs showed no growth in CSF, in contrast with wt meningococci (Fig. 2). After prolonged incubation in CSF, meningococci overexpressing NmsRs did start to grow (Fig. 2). However, sequence analysis of the region encoding NmsRs showed part of *nmsR*_B_ to be deleted in the escape variant.

### TCA cycle enzymes of *N. meningitidis* under the control of NmsRs.

To further assess the biological significance of the NmsRs, we compared the protein expression profile of the wt strain with that of the Δ*nmsR*_A_ Δ*nmsR*_B_ strain by mass spectrometric analysis of whole-cell lysates. Transcription of flanking genes of the Δ*nmsR*_A_ Δ*nmsR*_B_ strain remained unaffected after replacement of *nmsR*_A_ and *nmsR*_B_ with the erythromycin resistance cassette (not shown). Of all 2,300 annotated open reading frames in the *N. meningitidis* MC58 genome (17), 515 proteins (22%) were identified at the protein level. Of these, 387 yielded reliable quantification of relative expression comparing wt and Δ*nmsR*_A_ Δ*nmsR*_B_ strains (see Table S1 in the supplemental material). Differentially expressed proteins ranged from 7-fold upregulated to 6-fold downregulated. Using a 1.5-fold up- or downregulation as a cutoff for differential expression, a total of 18 upregulated and 10 downregulated proteins were identified (Table 1). Among the 18 proteins with increased expression in the Δ*nmsR*_A_ Δ*nmsR*_B_ strain, 10 (56%) were either involved in the TCA cycle directly or linked to it, such as acetate kinase (AckA-1), involved in acetyl coenzyme A (CoA) synthesis, or PrpC and PrpB, involved in propanoate metabolism feeding into the TCA cycle through succinyl-CoA. Other upregulated proteins belonged to the glycine cleavage system (GlyA), part of glycine/serine metabolism, or were involved in valine, leucine, and isoleucine degradation (3-hydroxyacid dehydrogenase; NMB1584). In contrast, proteins involved in ATP synthesis-coupled proton transport (AtpG and AtpC), a protein involved in the pentose pathway (Zwf), and proteins involved in biosynthesis of valine/leucine and isoleucine (IlvD and IlvA) are downregulated without NmsRs (Table 1). Complementation of the Δ*nmsR*_A_ Δ*nmsR*_B_ strain with a plasmid encoding both NmsRs led to normalization of protein levels for a slight majority of the overexpressed proteins identified (10/18; results not shown). Together, these results strongly suggest that in meningococci without NmsR activity, metabolism has been switched to higher TCA cycle activities, which are less strongly coupled to respiration. As we also observed notable expression of the NmsRs in transcriptome analyses of meningococci grown in nutrient-rich medium, this implies relatively low TCA cycle activity in meningococci grown in media with abundant nutrients. In the absence of NmsRs, the role of the TCA cycle in meningococcal metabolism increases, shifting to anabolism with, e.g., breakdown products of branched-chain amino acids as anaplerotic substrates and synthesis of components beneficial for growth under nutrient-poor conditions (Table 1).

10.1128/mBio.02293-16.2 TABLE S1  Proteins quantified by LC-MS^E^ in H44/76 wild-type and H44/76 Δ*nmsR*_A_ Δ*nmsR*_B_ strains. ^a^Mean of Hi3 peptide protein quantitation in normalized femtomoles for the biological replicates. ^b^Standard deviation expressed in percentage of the mean. ^c^Frequency of detection in 4 biological replicates. Download TABLE S1, TXT file, 0.2 MB. Copyright © 2017 Pannekoek et al. 2017 Pannekoek et al. This content is distributed under the terms of the Creative Commons Attribution 4.0 International license.

**TABLE 1:**
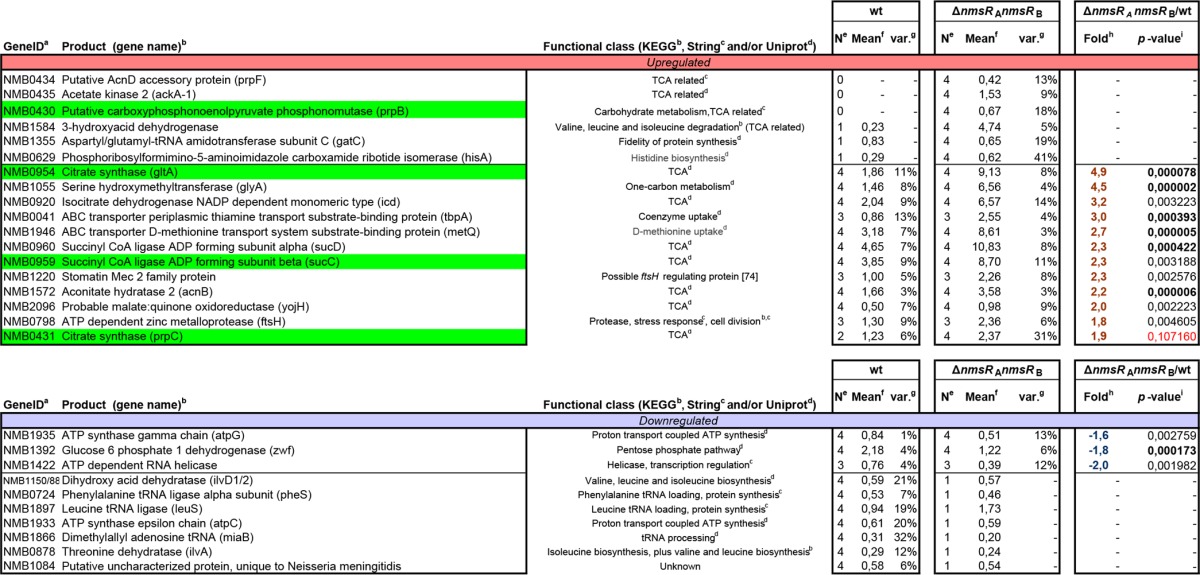
Differentially (≥1.5-fold change) regulated proteins in *N. meningitidis* Δ*nmsR*_A_ Δ*nmsR*_B_ strain as identified by LC-MS^E^

^a^ GeneID according to the work of Tettelin et al. (17).

^b^ Product and protein name according to KEGG (http://www.genome.jp/kegg/).

^c^ String (http://string-db.org).

^d^ UniProt (http://uniprot.org/uniprot).

^e^ Frequency of detection in 4 biological replicates.

^f^ In normalized attomoles.

^g^ Variance of standard error expressed in percent.

^h^ Fold change of ≥1.5. Downregulation is expressed as the reciprocal with added “−.”

^i^ Independent *t* test, two-tailed, equal variable. All samples, *P* ≤ 0.005 (except in red); bold values are significant after correction for false discovery rate according to the work of Benjamini and Hochberg (66). Genes indicated in green are confirmed as true targets of NmsRs in the *gfp* reporter system.

### NmsR_A_ and NmsR_B_ translational downregulation of TCA cycle enzymes is mediated by anti-Shine-Dalgarno sequences.

**FIG 3:**
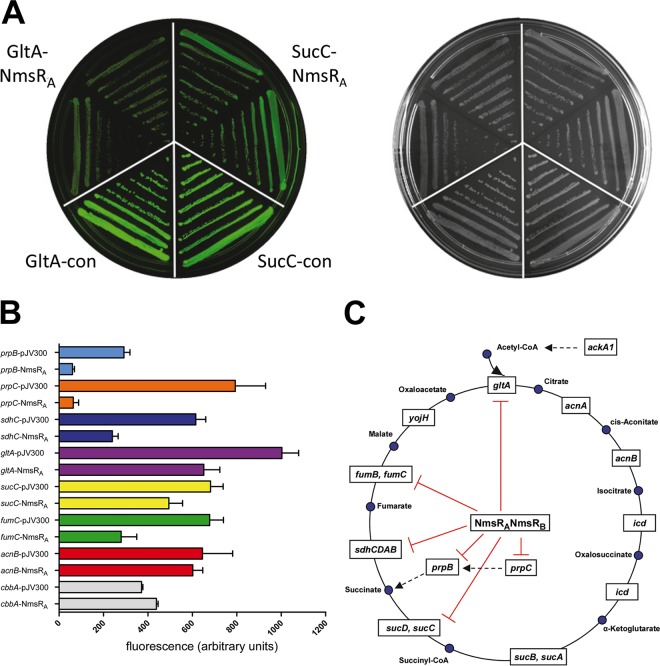
NmsR_A_ regulation of target mRNA expression. (A) Repression of translational fusions *gltA*::*gfp* and *sucC*::*gfp* by NmsR_A_. Shown are images of LB agar plates of *E. coli* carrying *gltA*::*gfp* fusion plasmid and a *sucC*::*gfp* fusion plasmid in combination with plasmid pJV300 (GltA-con and SucC-con) or pNmNmsR_A_ (GltA-NmsR_A_ and SucC-NmsR_A_) obtained in the fluorescence mode (left) or the visible light mode (right). Reduced colony fluorescence of the *gltA*::*gfp* or *sucC*::*gfp* fusion strains upon NmsR_A_ coexpression indicates regulation at posttranscriptional level. (B) **S**pecific regulation of target fusions by coexpression of NmsR_A_. Quantification of specific fluorescence signals from cells harboring combinations of fusion plasmids pJV300 and pNmNmsR_A_ as indicated. Error bars show standard deviations from experiments performed in quadruplicate. (C) Schematic representation of the TCA cycle with confirmed mRNA targets of NmsR_A_. Enzymes are shown within boxes, and metabolites are shown as blue dots. Red lines (inhibitory signals) denote confirmed NmsR_A_-downregulated target-*gfp* fusions (see text and panel B).

Results indicate that mRNAs encoding TCA cycle enzymes are potential targets for the NmsRs. *In silico* analysis (19) indeed revealed putative interactions between both NmsRs and 5′ untranslated regions (UTRs) of PrpB, PrpC, GltA, and SucC mRNAs. In addition, SdhC and FumC were identified as putative targets of NmsRs (Fig. S1 in the supplemental material). To obtain experimental evidence for the interaction between the NmsRs and potential target mRNAs, we used a well-established *gfp* reporter system in *Escherichia coli* (20). The 5′ UTR of the potential target mRNA and its first 7 to 13 codons were fused in frame to a *gfp* coding region (target-*gfp* fusion) which is constitutively expressed in *E. coli* together with the NmsRs from another plasmid vector. However, transformation of *E. coli* with plasmids harboring both *nmsR*_A_ and *nmsR*_B_ or only *nmsR*_B_ failed, even when a strain (JVS-2001) was used in which the sRNA chaperonin gene *hfq* was deleted or when a low-copy-number vector was used (20). However, *E. coli* could be transformed with the plasmid harboring only *nmsR*_A_, though it displayed attenuated growth (not shown) in all cases. Reduced fluorescence of target-*gfp* fusion in the presence of NmsR_A_ expression, but not in the presence of expression of a control nonsense sRNA, indicates a direct interaction between NmsR_A_ and the 5′ UTR of the target mRNA. In this way, direct translational control by NmsR_A_ was demonstrated for six out of eight tested putative target mRNAs (*prpB*, *prpC*, *sdhC*, *gltA*, *sucC*, and *fumC* [*P* < 0.005]) (Fig. 3A and B). The observation that fluorescence levels of the target-*gfp* fusion of two putative mRNA targets (*acnB* and *cbbA*) remained unaffected upon NmsR_A_ expression in *trans* indicates that the slower-growth phenotype of *E. coli* upon NmsR_A_ expression is not interfering with expression and/or proper folding of green fluorescent protein (GFP) as such (Fig. 3B).

10.1128/mBio.02293-16.1 FIG S1  Sequence conservation of the 5′ UTRs of putative target mRNAs of NmsR_A_ indicated by genome locus and functional annotation (17). (a) Nucleotides of 5′ UTRs of mRNAs shown to be targeted by NmsR_A_ are aligned with each other. Nucleotides complementary to putative α-SD sequences of NmsR_A_ are in green, G⋅U pairs in dark blue, and mismatches in red, and AUG initiations codons are underlined. Predicted stem-loop regions of NmsR_A_ (SL1 and SL2) are shown in black, α-SD1 and α-SD2 regions are shown in light blue, and the single-stranded region used for nucleotide alignments is shown in gray. Regions of NmsR_A_ used for alignment with target 5′ UTRs are shown in similarly colored lines adjacent to the predicted secondary structure of NmsR_A_ above the alignment. (b) Sequence Web-logo (68) of target 5′ UTRs listed in Fig. 2A. Download FIG S1, PDF file, 0.1 MB. Copyright © 2017 Pannekoek et al. 2017 Pannekoek et al. This content is distributed under the terms of the Creative Commons Attribution 4.0 International license.

Sequence comparison of the 5′ UTR of the target mRNAs with proven direct NmsR_A_ interaction showed homology around the Shine-Dalgarno sequence motif (SD), part of the ribosome binding site (Fig. S1) (21). The NmsRs are predicted to fold into similar secondary structures consisting of three stem-loops (SLs) (Fig. 1C). The single-stranded region between SL1 and SL2 exposes a UC-rich sequence. This region together with the UC-rich single-stranded loop of SL2 is (partly) complementary to the SD of the target mRNAs (Fig. S1). Both of these regions, referred to as α-SDs, are completely conserved among more than 7,335 meningococcal genomes analyzed (accessed at http://pubmlst.org/neisseria/) (16). Mutagenesis of either of the α-SD sequences in NmsR_A_ (using mutations designed to preserve the secondary structure of the NmsRs) abrogated the downregulation of all targets but GltA (Table S2). For the latter, mutagenesis of α-SD1 did not influence downregulation, but downregulation of GltA disappeared upon α-SD2 mutation (Table S2). Mutations outside α-SD regions had no effect on regulation (not shown). Replacement of nucleotides in the SD regions of the target mRNAs resulted in fluorescence levels of the cells below the level of detection. Consequently, it was not possible to investigate whether downregulation could be restored by the introduction of compensatory mutations. Taken together, these results strongly argue that NmsR_A_ inhibits synthesis by an antisense mechanism that involves direct base pairing to 5′ UTRs of six out of eight target-*gfp* fusions assessed, presumably by preventing ribosomal entry.

10.1128/mBio.02293-16.3 TABLE S2  Involvement of α-SD regions of NmsR_A_ in regulation of target fusions. ^a^Target-*gfp* fusion plasmid. ^b^Fold regulation observed with pNmNmsR_A_ (pJV300/pNmNmsR_A_). ^c^*P* value of significance of difference in regulation between pJV300 and NmsR_A_ or pJV300 and mutant NmsR_A_. ^d^Fold regulation observed with pNmNmsR_A_mα-SD1 (TCC^32−34^→CGA^32−34^) (pJV300/pNmNmsR_A_mα-SD1). ^e^Fold regulation observed with pNmNmsR_A_mα-SD2 (TCC^43−45^→CGA^43−45^) (pJV300/pNmNmsR_A_mα-SD1). Download TABLE S2, DOC file, 0.04 MB. Copyright © 2017 Pannekoek et al. 2017 Pannekoek et al. This content is distributed under the terms of the Creative Commons Attribution 4.0 International license.

### NmsRs alter expression of transcript levels of TCA cycle enzymes in meningococci.

**FIG 4:**
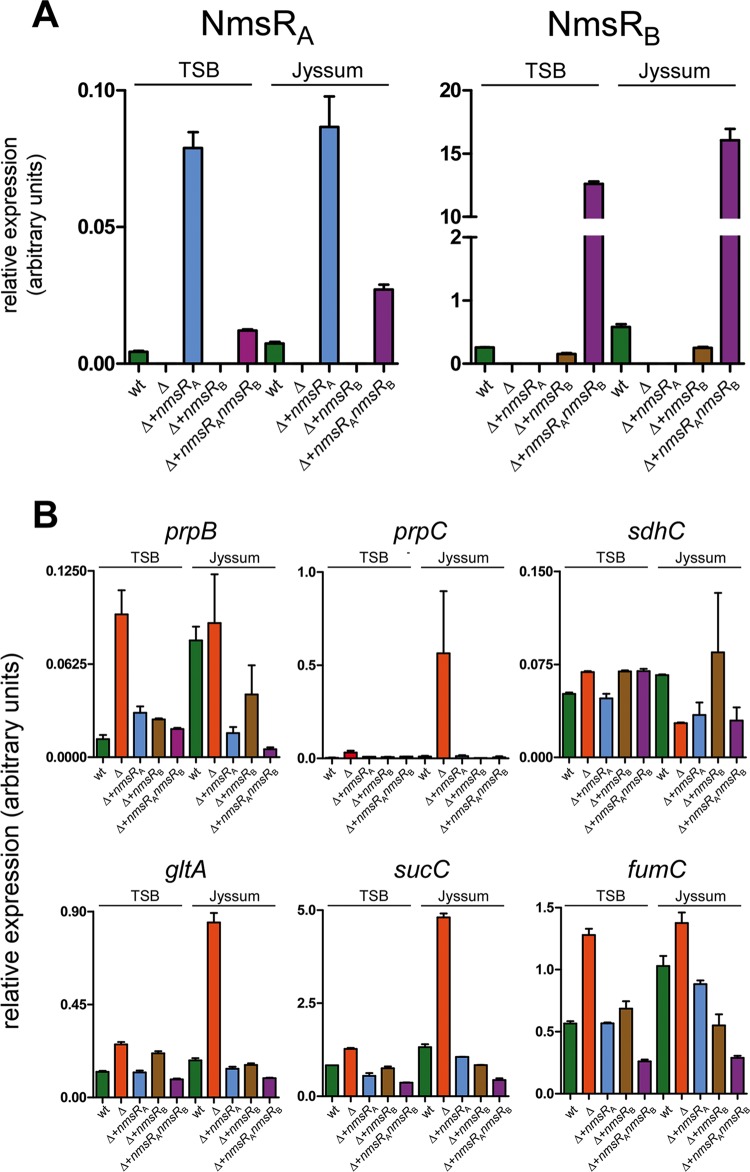
Transcript levels of TCA cycle enzymes in meningococci are under the control of NmsRs. (A) Relative expression levels of NmsRs. Transcript levels assessed by RT-qPCR in wt meningococci, in meningococci in which *nmsR*_A_ and *nmsR*_B_ are deleted (Δ) and in the Δ strain overexpressing NmsR_A_ (Δ+*nmsR*_A_), overexpressing NmsR_B_ (Δ+*nmsR*_B_), or overexpressing *nmsR*_A_ and *nmsR*_B_ (Δ+*nmsR*_A_*nmsR*_B_) (error bars, standard errors of the means; technical replicates, *n* = 8, over biological, *n* = 3). Meningococci were cultured in TSB (nutrient rich) and Jyssum medium (glucose as the sole carbon source [18]). (B) Relative expression levels of NmsR targets. Transcript levels assessed by RT-qPCR in wt meningococci and in meningococci in which *nmsR*_A_ and *nmsR*_B_ are deleted (Δ) and in the Δ strain overexpressing NmsR_A_ (Δ+*nmsR*_A_), overexpressing NmsR_B_ (Δ+*nmsR*_B_), and overexpressing *nmsR*_A_ and *nmsR*_B_ (Δ+*nmsR*_A_*nmsR*_B_) (error bars, standard errors of the means; technical replicates, *n* = 8, over biological, *n* = 3). Meningococci were cultured in TSB (nutrient rich) and Jyssum medium (glucose as the sole carbon source [18]).

The effect of NmsR expression on the expression of genes of the TCA cycle targeted by NmsR_A_ was assessed in meningococci grown under two culture conditions, TSB (nutrient rich) and Jyssum medium (glucose as the sole carbon source [18]), in which we anticipated differential expression. Transcript levels of all NmsR_A_ targets were indeed (1.5- to 8-fold) higher in meningococci with the NmsRs deleted and grown in TSB than in wt (*P* < 0.005). In Jyssum medium, transcript levels of *prpC*, *gltA*, and *sucC* were (5- to 3-fold) higher (*P* < 0.001) in the Δ*nmsR*_A_ Δ*nmsR*_B_ strain than in the wt strain. Transcript levels of *prpB* and *fumC* in Δ*nmsR*_A_ Δ*nmsR*_B_ and wt strains were not significantly different in Jyssum medium (Fig. 4). Of note, in all cases (except *sdhC*), the transcript levels of the targets were significantly lower in meningococci overexpressing NmsR_A_ (*P* < 0.05) or NmsR_B_ (*P* < 0.01) (or 2-fold lower in the case of *prpB* [*P* = 0.26]) or after overexpressing both sRNAs (*P* < 0.01) in the NmsR_A_ and NmsR_B_ deletion strain and in all these cases (except *sdhC*) became comparable to target levels found in wt meningococci when grown in TSB (Fig. 4). Transcript levels of *sdhC* in Jyssum medium were not significantly different in the wt strain from those in the NmsR_A_ and NmsR_B_ deletion strain of overexpression isogenic variants (Fig. 4).

### NmsRs are connected to the stringent response and controlled by RelA.

**FIG 5:**
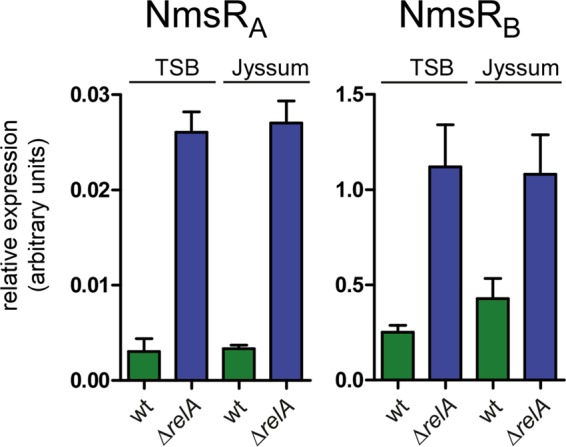
NmsR_A_ and NmsR_B_ levels in meningococci are under the control of the stringent response. Relative expression levels of NmsR_A_ and NmsR_B_, assessed by RT-qPCR, in wt meningococci and in meningococci in which *relA* is deleted (Δ*relA*) after growth in TSB (nutrient rich) and Jyssum medium (glucose as the sole carbon source [18]) (error bars, standard errors of the means; technical replicates, *n* = 8, over biological, *n* = 3).

In *Neisseria gonorrhoeae*, RelA is the sole producer of (p)ppGpp (22), which acts with DskA in interacting with RNA polymerase to regulate transcription. Whether a given promoter is directly controlled by (p)ppGpp and DksA is dictated by a DNA sequence motif, the so-called discriminator. Repressed targets typically contain GC-rich 7-nucleotide discriminators between the −10 box hexamer and the transcriptional start site, whereas activated promoters harbor AT-rich discriminators at this position (3). Of note, such a GC-rich nucleotide region can be identified between the putative −10 site and the transcriptional start site of NmsR_A_ (Fig. 1A). To investigate whether NmsRs are directly controlled by the stringent response, we created a *relA*-knockout strain of H44/76 (the Δ*relA* strain) by replacing *relA* with an erythromycin resistance cassette and assessed NmsR_A_ and NmsR_B_ levels after growth in TSB or Jyssum medium by reverse transcription-quantitative PCR (RT-qPCR). We did not obtain viable meningococci when *relA* was expressed in *trans*. Of interest, upon deletion of *relA*, NmsR_A_ transcript levels were 10-fold higher than wt levels (*P* < 0.0001), reaching levels that were comparable to NmsR_B_ levels in wt cells grown in TSB. NmsR_B_ levels were also significantly higher in Δ*relA* cells and increased 5- (*P* < 0.0005) and 2.5-fold (*P* < 0.05) in TSB and Jyssum medium, respectively (Fig. 5). No significant difference in levels of NmsRs was observed between cells grown in medium with glucose as sole carbon source and cells grown in nutrient-rich medium (Fig. 5).

**FIG 6:**
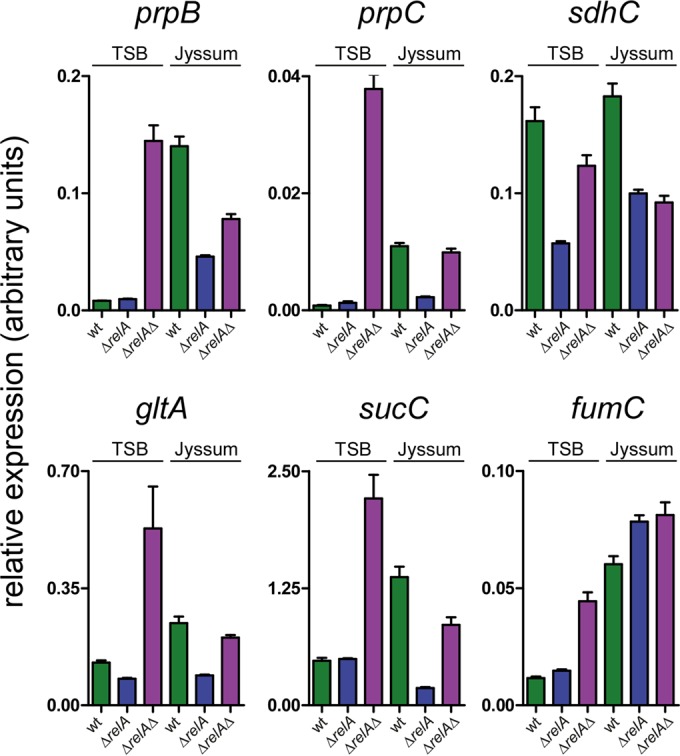
Transcript levels of NmsR targets are under the control of the stringent response by *relA*. Relative expression levels of NmsR targets, assessed by RT-qPCR, in wt meningococci, in meningococci in which *relA* is deleted (Δ*relA*), and in meningococci in which *relA nmsR*_A_, and *nmsR*_B_ are deleted (Δ*relA*Δ) after growth in TSB (nutrient rich) and Jyssum medium (glucose as the sole carbon source [18]) (error bars, standard errors of the means; technical replicates, *n* = 5, over biological, *n* = 1).

We next investigated whether transcript levels of the NmsR_A_ targets were affected upon deletion of *relA*. Transcript levels of all NmsR_A_ targets except *sdhC* in cells grown in TSB were relatively low and comparable in wt and Δ*relA* cells. In Jyssum medium, the transcript levels of all targets in the wt strain, again with the exception of *sdhC*, were 2- to 17-fold higher (*P* < 0.0005) than levels in cells cultured in TSB (Fig. 6). However, upon deletion of *relA*, transcript levels of *prpB*, *prpC*, *gltA*, and *sucC* were inversely correlated with levels of NmsR_A_ and NmsR_B_. In Jyssum medium, the transcript levels of the targets in Δ*relA* meningococci were 2- to 7-fold lower (*P* < 0.0001) than in wt meningococci and comparable to levels in wt cells or Δ*relA* cells grown in TSB (Fig. 6). Upon deletion of *nmsR*_A_ and *nmsR*_B_ in the Δ*relA* strain (Δ*relA* Δ*nmsR*_A_ Δ*nmsR*_B_) in cells cultured in TSB, the transcript levels of all targets, with the exception of *sdhC*, were 2- to 10-fold higher than those in Δ*relA* and wt strains. Of note, transcript levels of *prpB*, *prpC*, *gltA*, and *sucC* in the triple mutant Δ*relA* Δ*nmsR*_A_ Δ*nmsR*_B_ cells grown in Jyssum medium were 2- to 5-fold higher (*P* < 0.0001) than in the single Δ*relA* mutant. These results confirmed *relA*-mediated downregulation of NmsRs, irrespective of the culture conditions used. Transcript levels of *sdhC* and *fumC* in the triple-knockout Δ*relA* Δ*nmsR*_A_ Δ*nmsR*_B_ strain remained unaffected in Jyssum medium compared to the Δ*relA* single knockout (Fig. 6).

## DISCUSSION

In this study, we identified novel sibling regulatory sRNAs of *N. meningitidis* that establish a connection between the stringent response and the riboregulatory network. Our data showed regulation of the TCA cycle activity by direct action of sibling sRNAs in *N. meningitidis*. The expression of NmsRs themselves is under the control of the stringent response via RelA. The activity of the sibling sRNAs is crucial when meningococci encounter different host environments with variable nutrient supplies, such as blood and CSF. High sibling sRNA expression allows replication and survival in blood but impairs growth in CSF. Whether this is solely due to differential expression of TCA cycle enzymes or whether other, so-far-unknown targets are involved is the subject of further study.

NmsRs are the first sibling sRNAs and only the third sRNA species in *Neisseria* for which target genes are experimentally confirmed (23). NrrF, a Fur-regulated sRNA, has been identified in meningococci (24, 25) and gonococci (26) and is upregulated under iron-depleted conditions. This sRNA has been shown to be involved in regulation of *sdhA*, belonging to the operon encoding the succinate dehydrogenase complex (*sdhCDAB*). Recently, an sRNA was identified in *N. gonorrhoeae* that acts in *cis* and influences antigenic variation of pilin (27). Other sRNAs, among them AniS in meningococci and FnrS in gonococci, are synthesized under oxygen limitation, but their targets remain elusive (28, 29). The same is true for a σ^E^-dependent sRNA that has been identified in *N. meningitidis* (15).

By employing proteomics, putative targets of the NmsRs were identified. We experimentally validated direct interaction for four mRNAs coding for enzymes belonging to the TCA cycle (*sdhC*, *gltA*, *sucC*, and *fumC*) and for two mRNAs encoding enzymes producing intermediates of the TCA cycle (*prpB* and *prpC*) (schematically represented in Fig. 3C). Of interest was our identification of *sdhC* as a direct target of NmsRs. *sdhC* is the first gene of the cluster *sdhCDAB*, coding for the succinate dehydrogenase complex. This complex, which generates fumarate from succinate during the TCA cycle, concomitantly feeds electrons to the respiration chain (30). As mentioned before, in meningococci, NrrF has been shown to be involved in the Fur-dependent regulation of genes belonging to this cluster (24, 25). Although we showed a direct interaction between NmsR_A_ and *sdhC* resulting in downregulation of GFP by using the *gfp* reporter system, in the genetic background of meningococci we did not observe regulation. Likewise, we were also not able to confirm regulation for FumC in meningococci. This might imply either that both mRNAs are not true targets of NmsR_A_ or that expression of these transcripts, in the genetic background of meningococci, is more complex, e.g., under the control of other regulators as well. The latter is not unlikely since it has been shown that transcript levels of *sdhC* are controlled by the two-component regulator MisR (31), and as mentioned above, *sdhA* levels are controlled by NrrF. If true, *sdhCDAB* might be the first example of a cistronic mRNA in meningococci that is subject to regulation by two different sRNA species as well as a two-component regulator.

We identified *prpB* and *prpC* as targets of NmsRs. It has been shown that expression of these genes enables survival of the meningococci in the “normal” habitat (i.e., the adult nasopharynx) by allowing utilization of propionic acid as a supplementary carbon source (32). Thus, use of propionate becomes crucial under conditions of nutrient limitation. These observations are nicely in line with our data, as overexpression of NmsRs under nutrient-limiting conditions (e.g., Jyssum medium or liquor) leads to growth arrest. Tightly regulating NmsR expression is an essential prerequisite to support growth under divergent *in vivo* conditions, exemplified by colonization of the nasopharynx and replication in CSF.

We convincingly demonstrated that the expression of at least four different mRNA species is controlled by NmsRs. This makes NmsRs, as far as we know, the first example of sibling sRNAs in *N. meningitidis* acting on multiple *trans*-encoded targets, thus rewiring interconnected transcriptional networks, possibly including the MisR and Fur regulon. The unexpected transcript levels of *sdhC* and *fumC* observed with some strain-growth condition combinations could reflect such complex regulation.

Many small RNAs are known to contain one single-stranded domain that is able to interact with multiple target mRNAs (33–37). Other sRNAs have several functional domains that base pair with different sets of target mRNAs (38–41). Using *in vivo* experiments, we demonstrated that NmsR_A_ represses synthesis of its mRNA targets most likely by an antisense mechanism. This involves base pairing of predicted single-stranded α-SD regions (UC-rich) of the NmsRs to a sequence stretch overlapping the SD in the targets. Basically, this antisense mechanism is shared by many other sRNAs (7, 42). Of interest, the NmsRs contain two α-SD regions apparently acting on the same set of mRNAs by duplex formation with the region encompassing the SD. Both α-SD regions are characterized by UC-rich stretches but differ slightly from each other in sequence. In five out of six cases, action of both α-SDs is required for downregulation, suggesting coordinated activity, while in one case (*gltA*) downregulation is abrogated only after mutating α-SD2. This suggests that, for this target, α-SD2 acts independently of α-SD1 and that only duplex formation with α-SD2 is essential. Calculation of the minimum free energy (MFE) of the duplexes before and after mutagenesis of the α-SDs using *RNAhybrid* (43) could not accurately predict the *in vivo* outcome of this regulation (not shown). However, similar modes of action have recently been described for the LhrC family of sibling sRNAs of *Listeria monocytogenes* (44, 45), and future experiments are necessary to investigate whether, for example, less conserved flanking sequences of the region of interaction of NmsRs with their targets might contribute to a different affinity and subsequent outcome of the duplex formation. The finding that *in silico* predictions of duplex formation based on complementarity of target and sRNA sequences do not always match *in vitro* observations, e.g., the predicted target *cbbA* apparently not being controlled by NmsR_A_, is also important in this context.

We observed that expression of both NmsRs or NmsR_B_ in *E. coli* did not result in viable cells, while expression of NmsR_A_ in *E. coli* showed attenuated growth. Possibly, the expression of both NmsRs is toxic for *E. coli*. Alternatively, *E. coli* encodes (an) NmsR_A_ and NmsR_B_ target(s), which will be interesting to identify as well. This interpretation is strengthened by the observation that the slower-growth phenotype of *E. coli* disappeared after mutagenesis of the α-SDs of NmsR_A_ (not shown).

The activity of many sRNAs in bacterial pathogens depends on the RNA chaperone Hfq (13, 14, 46). Among the direct targets of NmsR_A_ identified, three proteins (GltA, PrpB, and PrpC) were also found to be overexpressed in an Hfq deletion strain of *N. meningitidis* (Δhfq) (47). This overlap between some of the NmsR_A_ targets and Hfq-dependent mRNAs might indicate that for these cases NmsRs act in concert with Hfq. In general, two signatures in the sequences of sRNAs are reported as preferred binding sites for Hfq. The first is a typical A/U-rich single-stranded stretch that precedes the predicted Rho-independent terminator. The second signature consists of terminal U residues (14, 48, 49). The second signature is obviously present, but the first signature seems absent from NmsRs. Thus, whether the observed overlap between the Hfq and NmsR_A_ regulon in *N. meningitidis* represents a joint action of the chaperone and sRNAs, or represents a more indirect phenomenon, awaits further experiments.

The continuous discovery of more sRNAs has resulted in the identification of several examples of homologous sRNAs, “sibling” sRNAs (50). We identified a novel sibling member of this expanding class of sRNAs. The NmsRs are encoded in tandem in an intergenic region. Equal expression levels of the two sRNAs were observed under nutrient-rich and nutrient-poor conditions, but relative expression levels of NmsR_A_ were very low compared to those of NmsR_B_. The relatively high expression levels of NmsR_B_ might suggest that NmsR_B_ acts redundantly in a compensatory manner on the same targets, as has been described for sRNAs of other pathogens (36, 51, 52). The system is more complicated, however, as illustrated by the fact that target levels were significantly downregulated upon overexpression of single NmsRs but expression of both was required for complete repression (demonstrating combined NmsR action). In addition, the action of both sRNAs was also required for growth inhibition of meningococci under nutrient-limiting conditions. Thus, these observations suggest classification of NmsRs as riboregulators that act cumulatively, each contributing in a different degree to overall adaptation. Homologous sRNAs acting together have also been described for other pathogens (50, 53). It should be noted that although single NmsR_A_ levels were low and single NmsR_B_ levels were significantly lower when they were expressed in Δ*nmsR*_A_ Δ*nmsR*_B_ cells, they were still sufficient to downregulate 5′ UTR *gfp* fusion products in *E. coli* and mRNA target levels in meningococci.

How the meningococcal NmsRs are regulated themselves and whether they are fine-tuned individually with regard to their own expression levels as well as their target preferences have to be elucidated further. We could show the expression of NmsRs to be elevated in a *relA* knockout, indicating that it is connected to the stringent response. A direct interaction of (p)ppGpp with the putative negative discriminator in the 5′ UTR of NmsR_A_ looks tempting. Of interest, many gammaproteobacterial genes shown to be direct targets of (p)ppGpp contain typical σ^70^-dependent promoters (3). Indeed, inspection of the predicted promoter region of NmsR_A_ shows a high similarity to σ-dependent promoters with the σ^70^ signatures identified in *E. coli* (−35 element TTGACA [*E. coli* consensus TTGACA] and −10 element GATAAT [*E. coli* consensus TATAAT]) (54–56). Also, the 5′ UTR of NmsR_B_ has an, albeit weaker, σ^70^ signature. This might suggest σ-dependent NmsR_A_ transcription directly controlled by (p)ppGpp. Expression levels of NmsR_B_ are much higher when the two single sRNAs are coexpressed, reflecting less restricted transcription for this sRNA. Cotranscription of the two sRNAs might also be needed for their stabilization. How the possible mutual stabilization and interregulation might work is under investigation. Alternatively, the NmsRs are indirectly controlled by (p)ppGpp. In some cases, regulators of sRNA expression are located in the close vicinity of the particular sRNA to be regulated (57). The gene encoding Lrp (NMB1650) is located directly downstream of the locus encoding the sibling sRNAs (though in opposite orientation). The expression of Lrp might be stimulated by (p)ppGpp (58). However, NmsR levels did not significantly change upon either deletion or overexpression of *lrp* (not shown).

In conclusion, we identified sibling sRNAs targeting genes encoding TCA cycle enzymes, stressing their importance in the adaptation to changing environments in the host. The riboregulated network of the sibling sRNAs is part of the RelA-regulated stringent response. NmsRs of *N. meningitidis* form a crucial part of the riboregulatory network monitoring metabolic status, translating this into colonization with likely implications for pathogenesis.

## MATERIALS AND METHODS

### Bacterial strains and culture conditions.

*N. meningitidis* strain H44/76, B:P1.7,16: F3-3:ST-32 (cc32), is closely related to the sequenced serogroup B strain MC58, belonging to the same clonal complex (59). Meningococci were grown for 16 to 18 h on GC plates (Difco) supplemented with 1% (vol/vol) Vitox (Oxoid) or on PVX plates (bioMérieux) at 37°C in a humidified atmosphere of 5% CO_2_. Broth culturing was performed in tryptic soy broth (TSB) (BD), GC medium supplemented with 1% (vol/vol) Vitox, or Jyssum medium (18), on a gyratory shaker (180 rpm) at 37°C. When appropriate, plates or broths were supplemented with erythromycin (Erm) (5 μg/ml) and/or chloramphenicol (Cm) (5 μg/ml) and/or kanamycin (50 μg/ml). Expression of recombinant DNA in meningococci was induced by IPTG (isopropyl-β-d-thiogalactopyranoside) (0.5 mM). Growth in broth was monitored by measuring optical density of cultures at 530 nm (OD_530_) at regular time intervals. Growth of meningococci in human blood and CSF was monitored as follows. Blood was collected in 4-ml Vacutainers with 17 IU/ml sodium heparin (BD) from a healthy male volunteer approximately 2 h prior to use. CSF was extracted, with informed consent, from patients with suspected normal-pressure hydrocephalus, either used fresh (within <24 h stored at 4°C) or aliquoted and stored at −80°C. CSF white blood cell count and glucose and protein levels were within normal range. Heparinized human blood and CSF, the latter diluted prior to use to 50% (vol/vol) with phosphate-buffered saline (PBS), was inoculated with the equivalent of approximately 1 × 10^5^ meningococci, which were obtained from precultures in TSB (OD_530_, ~0.2 to 0.4). Aliquots of 220 µl were incubated in sterile 96-well plates (Corning) and incubated at 37°C in a humidified atmosphere of 5% CO_2_. At regular time intervals, 20-µl samples were serially diluted and plated on PVX plates (bioMérieux), and colonies were counted after 16 to 18 h of growth at 37°C in a humidified atmosphere of 5% CO_2_.

*E. coli* strain Top10 (Invitrogen) was used to clone *gfp* fusions and in experiments involving coexpression of *gfp* fusions and sRNAs. *E. coli* strain Top10F′ (Invitrogen) was used to clone sRNA expression plasmids and pCR2.1 (Invitrogen) and pEN11-*pldA* (60) constructs. *E. coli hfq*-knockout strain JVS-2001 was kindly provided by J. Vogel (Würzburg, Germany). *E. coli* strains were grown in lysogeny broth (LB) (Oxoid) (2% [wt/vol] in distilled water [dH_2_O]) or on LB agar (1% [wt/vol]) plates. Liquid *E. coli* cultures were grown in 5 ml of medium inoculated from a single colony overnight at 37°C on a gyratory shaker (250 rpm). Antibiotics were applied to *E. coli* cultures at concentrations of 100 μg/ml (ampicillin) and 20 μg/ml (chloramphenicol).

Plasmid DNA from *E. coli* was isolated from overnight cultures grown in LB using the Wizard Plus SV Minipreps DNA kit (Promega). PCRs were performed according to standard protocols using a Biometra PCR machine. Primer sequences are listed in Table S3 in the supplemental material. DNA was gel purified using a GeneJET gel extraction kit (Thermo Scientific). Digestion and ligation were carried out using enzymes supplied by New England BioLabs or Thermo Scientific. Plasmid pCR2.1 was used for cloning and sequencing of PCR products. Plasmids pXG-0 (control for autofluorescence), pXG-1 (control for sRNA effect on *gfp* expression), and pXG-10 (standard plasmid for *gfp* fusion cloning) were kindly provided by J. Vogel (Würzburg, Germany) and have been described previously (20). The *nmsR*_A_ gene was amplified using primers RHsRNA25CFW11 and RHsRNA25GFPRV13b and inserted into the sRNA-expressing plasmid pZE12-*luc*, thereby creating pNmNmsR_A_ using the cloning strategy described previously (20). The shuttle vector pEN11-*pldA* was used to express sRNAs in meningococci (60).

10.1128/mBio.02293-16.4 TABLE S3 Oligonucleotides used in this study. Sequences are given in 5′-to-3′ direction. “5-P” denotes 5′ phosphorylation. Restriction sites are underlined. Download TABLE S3, TXT file, 0.02 MB. Copyright © 2017 Pannekoek et al. 2017 Pannekoek et al. This content is distributed under the terms of the Creative Commons Attribution 4.0 International license.

*N. meningitidis* was transformed as described previously (61). Transformants were plated on selective plates containing appropriate antibiotics and checked by PCR for integration and orientation of the erythromycin or kanamycin resistance cassette. All constructs were verified by Sanger sequencing.

### Fluorescence measurements of *gfp E. coli* reporter strains and data processing.

*E. coli* Top10 cells expressing *gfp* fusions were streaked on standard LB plates supplemented with appropriate antibiotics. After overnight growth, colonies were photographed in a Syngene Bio Image analyzer using a Lumenera camera with a 510-nm emission filter and excitation at 460 nm. Fluorescence measurements in 96-well plates were carried out as described previously (20). In brief, single colonies (in quadruplicate) of *E. coli* strains harboring a target-*gfp* fusion and sRNA-expressing plasmids were inoculated in 200 μl LB in a 96-well microtiter plate, and cultures were grown at 37°C. The OD was measured at 600 nm in an enzyme-linked immunosorbent assay (ELISA) reader (Anthos Labtec), and fluorescence was measured (optical excitation filter, 485/20 nm; emission filter, 530/25 nm) in a CytoFluor II multiwell plate reader (PerSeptive Biosystems). The linear range of increasing fluorescence during growth covered by all members of a quadruplicate was selected to obtain the specific fluorescence. To calculate the specific fluorescence, the total fluorescence of a given strain expressing NmsR_A_ and a target-*gfp* fusion gene (the mean fluorescence of the quadruplicate at a chosen time point within the linear range) was corrected for the autofluorescence measured in strains harboring an NmsR_A_ expression plasmid or control sRNA in combination with the negative-control plasmid pXG-0 (expressing luciferase, i.e., no *gfp*). The regulatory effect of NmsR_A_ on a target-*gfp* fusion was expressed as fold regulation (mean of the quadruplicate values). This is calculated by dividing the unregulated *gfp* fusion specific fluorescence (negative-control sRNA pJV300) by the regulated *gfp* fusion specific fluorescence (sRNA of interest) (20).

### Construction of Δ*nmsR*_A_ Δ*nmsR*_B_ and Δ*relA* mutants of *N. meningitidis* strain H44/76.

*N. meningitidis* H44/76 knockout mutants of *nmsR*_A_ and *nmsR*_B_ and of *relA* (NMB1735) were constructed using the PCR-ligation-PCR method as described previously (62). PCR products were generated with primer pairs YPsRNA25FWKO1-YPsRNA25RPKO2 and YPsRNA25FWKO3-YPsRNA25RPKO4 to create the Δ*nmsR*_A_ Δ*nmsR*_B_ strain and primer pairs KSrelAF1-KSrelAR2 and KSrelAF6-KSrelAR7 to create the Δ*relA* strain and ligated. The ligation products were reamplified with primer pair YPsRNA25FWKO1-YPsRNA25RPKO4 (for *ΔnmsR*_A_ *ΔnmsR*_B_) and primer pair KSrelAF1-KSrelAR7 (for Δ*relA*). Resulting PCR products were cloned into pCR2.1 (Invitrogen). The EcoRI-digested Erm resistance cassette from pAErmC′ was introduced into the created unique MfeI restriction site, yielding plasmids pCR2.1-sibling sRNA and pCR2.1-NMB1735 (*relA*) (62). The Δ*nmsR*_A_ Δ*nmsR*_B_ and Δ*relA* knockout strains were generated by natural transformation of strain H44/76 with pCR2.1-NmsR_A_NmsR_B_ and pCR2.1-NMB1735, respectively, with selection for Erm resistance. Replacement of NmsR_A_ and NmsR_B_ and NMB1735 by the Erm cassette was confirmed by PCR with primer pairs that were used for amplification of the ligation products and sequence analysis. Mutant strains in which the transcriptional direction of the Erm cassette was in accordance with the original transcriptional direction of the deleted genes were selected. To create the Δ*relA* Δ*nmsR*_A_ Δ*nmsR*_B_ triple-knockout strain, the same strategy as that described for the creation of single knockouts was used, but in this case, the *relA* gene in the Δ*nmsR*_A_ Δ*nmsR*_B_ strain was replaced by a kanamycin resistance cassette. The kanamycin resistance cassette was amplified using plasmid pDOC-F as the template and primer set pDOCF1 and pDOCF2 (63), and primer KSrelAF6-P in combination with primer KSrelAF7 was used instead of primer KSrelAF6. Transcription of flanking genes of the knockout strains was controlled by RT-qPCR and remained unaffected.

### Overexpression of *nmsR*_A_ and *nmsR*_B_ and *relA*.

The construction of plasmids overexpressing sRNAs was carried out as described previously (60). In brief, for *nmsR*_A_ and *nmsR*_B_ together and separately, the regions encoding both NmsR_A_ and NmsR_B_, only NmsR_A_, and only NmsR_B_ of strain H44/76 were amplified with primer pairs CT_sRNA25FW/CT_sRNA25Rev (for both NmsR_A_ and NmsR_B_), RHsRNA25CFW11/RHsRNA25CRV13b (for NmsR_A_ only), and RHsRNA25CFW12/RHsRNA25CRV13b (for NmsR_B_ only). Reverse primers contained an RcaI site. Primer pair YPpen11MauB1-YPpen11plus1, with pEN11-*pldA* as the template, was used to generate a part of pEN11-*pldA* containing a MauBI restriction at the 3′ end and the promoter sequence and the region between the −10 box and the transcriptional start of the farthest part of the 5′ end. This fragment was ligated to the PCR products encoding both NmsR_A_ and NmsR_B_ or NmsR_A_ and NmsR_B_ separately, and the ligation product was PCR amplified using primer pair YPpen11MauB1-CT_sRNA25Rev (for NmsR_A_ and NmsR_B_ together) and primer pair YPpen11MauBI-RHsRNA25CRV13b (for NmgR_A_ and NmsR_B_ separately). The resulting PCR products and pEN11-*pldA* were digested with MauBI and BspHI, ligated into MauBI- and BspHI-predigested pEN11-*pldA*, and transformed to *E. coli* Top10F′ (Invitrogen). Cm-resistant colonies were checked by colony PCR and sequencing, using pEN11FW2 and pEN11R primers. Plasmids of clones containing an intact coding region for both NmsR_A_ and NmsR_B_ (pEN11_NmsR_A_NmsR_B_) or an intact NmsR_A_ or NmsR_B_ (pEN11_NmsR_A_ or pEN11_ NmsR_B_, respectively) were isolated to transform H44/76, thereby generating H44/76*+*pEN11_NmsR_A_NmsR_B_, H44/76+pEN11_NmsR_A_, and H44/76+pEN11_NmsR_B_, respectively. Because transformation of cells (*E. coli* or meningococci) with constructs overexpressing *relA* did not yield viable cells, we did not succeed in creating a strain overexpressing *relA*.

### *In vitro* mutagenesis of *nmsR*_A_.

Construct pNmNmsR_A_ was used to generate mutant sNmsR_A_s. Point mutations were generated using QuikChange site-directed mutagenesis (Stratagene). Two mutants of pNmNmsR_A_ in α-SD regions were generated, pNmNmsR_A_mα-SD1 and pNmNmsR_A_mα-SD2, using primer pair RHsR25A1eSD_F-RHsR25A1eSD_R (for mutant pNmNmsR_A_mα-SD1) and primer pair RHsR25A2eSD_F-RHsR25A2eSD_R (for mutant pNmNmsR_A_mα-SD2). Two mutants of pNmNmsR_A_ outside α-SD regions were generated, pNmNmsR_A_mLoop3 and pNmNmsR_A_m2Loop, using primer pair YPsR25Loop3_F-YPsR25Loop3_R (for mutant pNmNmsR_A_mLoop3) and primer pair RHsR25A2Loop_F-RHsR25A2Loop_R for pNmNmsR_A_m2Loop). Mutations were confirmed by sequence analysis.

### WTA and RT-qPCR.

WTA was carried out as described previously (15). For RT-qPCR, RNA was extracted from meningococci grown to log phase (OD_600_, 0.2 to 0. 5) using the miRNeasy minikit (Qiagen) followed by Turbo DNase Turbo DNA-free kit (Life Technologies) treatment. Then, cDNA was synthesized from 1.5 µg of RNA and random oligonucleotide hexamers using ThermoScript reverse transcriptase (RT) (Invitrogen) according to the manufacturer’s recommendations. Quantitative PCR was performed using LightCycler 480 SYBR Green I Master in the LightCycler 480 system (Roche). The identities of the resulting amplicons were checked by melting-curve analysis using the LightCycler 480 and 1.5% agarose gel electrophoresis. Reaction mixtures containing no template were included in each real-time PCR experiment to control for contamination. Transcripts of target and reference genes were analyzed using LinRegPCR version 2014.2 (64). Constitutive relative gene expression in medium was determined as a ratio of target gene to reference genes (*rmpM* [NMB0382] and *cbbA* [NMB1869]).

### Sample preparation and mass spectrometric analysis.

Cells from mid-logarithmic-phase Δ*nmsR*_A_ Δ*nmsR*_B_ and wt strains were rapidly cooled and harvested by centrifugation. Cell pellets were resuspended in lysis buffer consisting of 0.1% RapiGest SF (Waters Corporation, Milford, MA) in 50 mM ammonium-hydrogen carbonate (pH 8.0) (Sigma-Aldrich) and lysed by sonication. The protein content of the different samples was determined by bicinchoninic acid assay (Thermo Scientific, Rockford, IL) using the manufacturer’s protocol. Overnight proteolysis of samples and subsequent removal of RapiGest surfactant were performed according to the protocol provided with RapiGest SF for in-solution digestion using a 1:50 (wt/wt) ratio of trypsin (Promega, Madison, WI) to protein. Peptide samples were then mixed 1:1 (vol/vol) with 100 nM ADH1 from *Saccharomyces cerevisiae* digest standard (Waters Corporation, Milford, MA) prior to separation by reversed-phase chromatography and analysis by data-independent (MS^E^) label-free mass spectrometry as described before (65) on a Synapt-G2 quadrupole time of flight mass spectrometer (Waters Corporation, Milford, MA). Continuum liquid chromatography (LC)-MS^E^ data were processed and searched using ProteinLynx GlobalSERVER version 2.5 (PLGS 2.5; Waters Corporation, Milford, MA). Parameter settings were as described in reference 65. Protein identifications were obtained by searching an *N. meningitidis* database (UniProt release 2012_03) with common protein contaminants, as well as ADH1 from *S. cerevisiae* as an internal standard, appended, to address technical variation and allow concentration determinations between different samples (65). The estimation of the false-positive identification rates was performed by searching a randomized version of the abovementioned *N. meningitidis* protein database generated within PLGS 2.5. Data were exported as csv-files for further, detailed analysis. Stringent criteria were applied for quantitation: protein identifications were considered significant only if reported in at least 2 out of 4 biological replicates. Protein false-positive identification rates estimated using this criterion were ~2.3% for a total of 533 identifications. To obtain quantitative information on protein expression in comparing Δ*nmsR*_A_ Δ*nmsR*_B_ and wt strains, the amounts in femtomoles estimated by PLGS 2.5 through HI3 peptide quantitation (65) were first normalized by the sum of all proteins quantified for each individual sample. Subsequently, the average of the normalized femtomoles from the 4 biological replicates was calculated if detected in >1 biological replicate and used to calculate the fold change between Δ*nmsR*_A_ Δ*nmsR*_B_ and wt strains if a value for both strains was obtained (see Table S1 in the supplemental material). If a change was ≥1.5-fold up- or downregulated, a *t* test was performed to ascertain whether the change in protein expression was significant, i.e., whether it had a *P* value of ≤0.05 following adjustment for multiple testing according to the method of Benjamini and Hochberg (66). Proteins which were detected in only one of the two strains were reported as uniquely detected only if all of the quadruplicate injections of the biological replicates of one strain yielded quantitative data for this protein while the protein was not detected in any injections of the other strain. When a protein was detected in all injections from one strain and also in one of the replicate injections of the other, the value in normalized femtomoles is given for that single detection (i.e., no fold change value), as this would give the impression that the protein was consistently detected in both strains (Table 1).

### Accession number(s).

The mass spectrometry proteomics data have been deposited in the ProteomeXchange Consortium (http://www.proteomexchange.org) via the PRIDE partner repository (67) with the data set identifier PXD000891.
