# Supplementary material for: Neisseria meningitidis Uses Sibling Small Regulatory RNAs To Switch from Cataplerotic to Anaplerotic Metabolism
Source: mBio. 2017 Mar 21;8(2):e02293-16. doi: 10.1128/mBio.02293-16 (PMC5362039; doi:10.1128/mBio.02293-16)
Supplement: FIG S1 [file mbo001173235sf1.pdf]

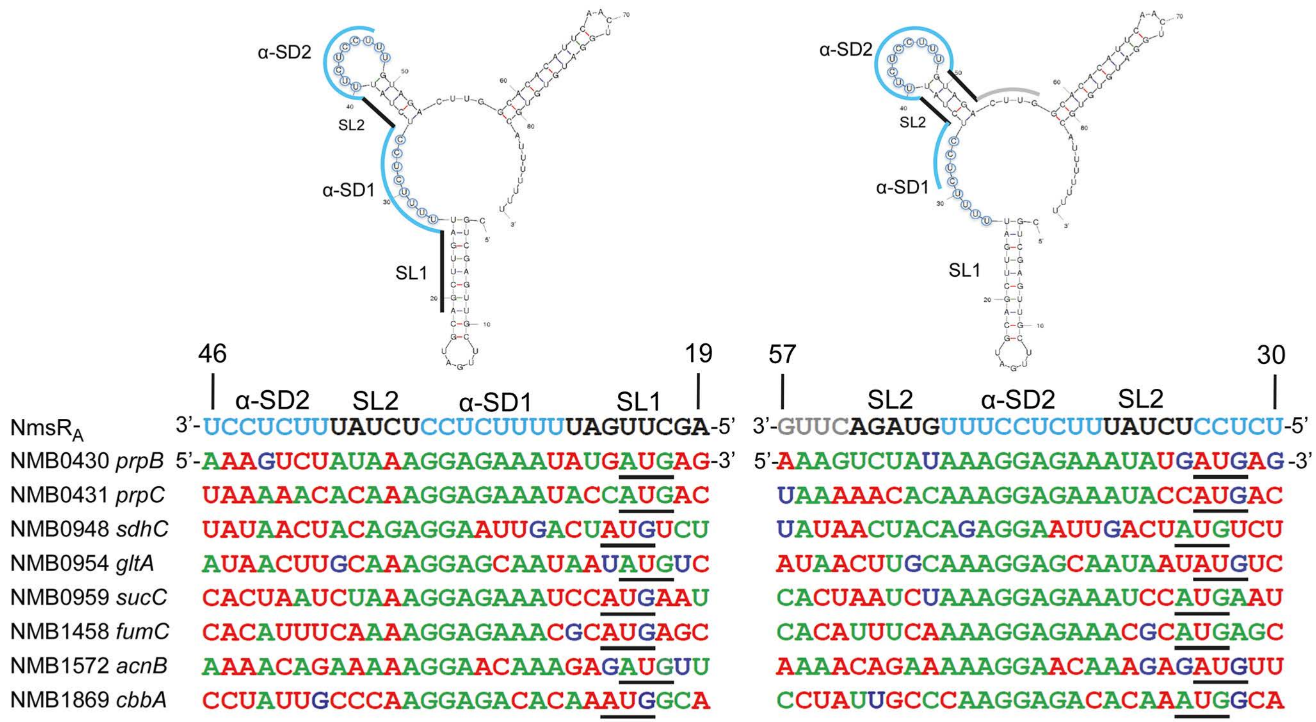

α-SD2
SL2
α-SD1
SL1
α-SD2
SL2
α-SD1
SL1
46
19
α-SD2 SL2 α-SD1 SL1
NmsRA 3'-UCCUCUUUAUCUCCUCUUUUUAGUUCGA-5'
NMB0430 prpB 5'-AAAGUCUAUAAAGGAGAAAUAUGAUGAG-3'
NMB0431 prpC UAAAAACACAAAGGAGAAAUACCAUGAC
NMB0948 sdhC UAUAACUACAGAGGAAUUGACUAUGUCU
NMB0954 gltA AUAACUUGCAAAGGAGCAAUAAUAUGUC
NMB0959 sucC CACUAAUCUAAAGGAGAAAUCCAUGAAU
NMB1458 fumC CACAUUUCAAAAGGAGAAACGCAUGAGC
NMB1572 acnB AAAACAGAAAAAGGAACAAAGAGAUGUU
NMB1869 cbbA CCUAUUGCCCAAGGAGACACAAAUGGCA
57
30
SL2 α-SD2 SL2
3'-GUUCAGAUGUUUCCUCUUUAUCUCCUCU-5'
5'-AAAGUCUAUAAAGGAGAAAUAUGAUGAG-3'
UAAAAACACAAAGGAGAAAUACCAUGAC
UAUAACUACAGAGGAAUUGACUAUGUCU
AUAACUUGCAAAGGAGCAAUAAUAUGUC
CACUAAUCUAAAGGAGAAAUCCAUGAAU
CACAUUUCAAAAGGAGAAACGCAUGAGC
AAAACAGAAAAAGGAACAAAGAGAUGUU
CCUAUUGCCCAAGGAGACACAAAUGGCA

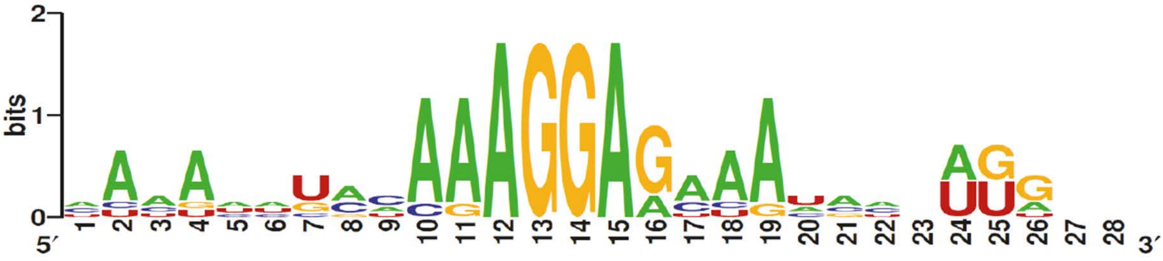

bits
0
1
2
5′
1 2 3 4 5 6 7 8 9 10 11 12 13 14 15 16 17 18 19 20 21 22 23 24 25 26 27 28
3′
