# Supplementary material for: Neisseria meningitidis Uses Sibling Small Regulatory RNAs To Switch from Cataplerotic to Anaplerotic Metabolism
Source: mBio. 2017 Mar 21;8(2):e02293-16. doi: 10.1128/mBio.02293-16 (PMC5362039; doi:10.1128/mBio.02293-16)
Supplement: TABLE S2 [file mbo001173235st2.doc]

**Additional file 3: Table S2**

Involvement of α-SDs regions of NmsR-A in regulation of target fusions.

| fusion^a^ | Wild-type^b^(*p*)^c^ | mα-SD1^d^(*p*)^c^ | mα-SD2^e^(*p*)^c^ |
| --- | --- | --- | --- |
| *prpB* | 4.9 (<0.0001) | 0.9 (0.546) | 0.7 (0.002) |
| *prpC* | 7.6 (<0.0001) | 0.8 (0.030) | 0.8 (0.076) |
| *sdhC* | 2.1 (<0.0001) | 1.0 (0.508) | 1.0 (0.772) |
| *gltA* | 1.5 (0.0005) | 1.5 (0.001) | 1.0 (0.700) |
| *sucC* | 1.4 (0.0042) | 1.0 (0.724) | 1.1 (0.406) |
| *fumC* | 1.8 (0.0002) | 0.9 (0.089) | 0.8 (0.001) |
|  |  |  |  |

^a^Target *gfp* fusion plasmid; ^b^Fold-regulation observed with pNmNmsR-A (pJV300/pNmNmsR-A); ^c^*P*-value of significance of difference in regulation between pJV300 and NmsR-A or pJV300 and mutant NmsR-A; ^d^Fold-regulation observed with pNmNmsR-Amα-SD1 (TCC^32-34^→CGA^32-34^) (pJV300/pNmNmsR-A mα-SD1); ^e^Fold-regulation observed with pNmNmsR-Amα-SD2 (TCC^43-45^→CGA^43-45^) (pJV300/pNmNmsR-A mα-SD1).
